# Is Quinine a Partus Accelerator?

**Published:** 1866-10

**Authors:** D. L. Phares

**Affiliations:** Newtonia, Mississippi


### ARTICLE II.

*Is Quinine a Partus Accelerator?* By D. L. Phares, M. D., Newtonia, Mississippi.

"There was a tendency to congestion, often from the first, almost always in progress; hence the value of this combination. In a case (a company laundress) of relapse, attended with rapid and feeble pulse, pungent heat of the skin, etc., quinine and capsicum were used freely, and in the course of eighteen hours, the patient was considered out of danger. Unfortunately, in about twenty-four hours from the second attack of fever, abortion of a three months' fœtus (with considerable hemorrhage) occurred, reducing the patient to an extreme condition. The combination of quinine and capsicum was steadily continued, in addition to ether, sulphuric brandy, etc., until the urgent symptoms passed off, and the patient recovered." *Report to Surgeon-General on Epidemic Fever on Sullivan's Island, in* 1850. By John B. Porter, M. D., Surgeon U. S. A.

While looking over the report quoted above, my eye incidentally caught the above lines, which attracted my attention, from the fact that, last night, my friend Dr. Carruth asked me if I had ever observed any cases of abortion follow the exhibition of quinine. Dr. W., of A., having observed several abortions occur in cases in which he had given quinine, directed his attention more especially to this subject, and finally became convinced that quinine induces abortion. Hence he abstains from its use during pregnancy. However, some months ago, having a case in which the intermittent became very annoying to a pregnant woman, and refused for many weeks to yield to other remedies, Dr. W. finally, with much reluctance, gave quinine. Abortion soon ensued. He "will never again give quinine to a pregnant woman."

Dr. P., of C., has repeatedly noticed a similar sequence of events, and hence positively refuses to exhibit quinine during pregnancy, declaring that it certainly induces abortion in his hands.

Several similar cases had recently come to the knowledge of Dr. Carruth. In one of these, Dr. C. exhibited quinine, and soon after, meeting Dr. P., told him what he had done. Dr. P. replied: "She will be sure to abort;" and abort she did, twenty-four hours after taking the medicine!

Dr. C. contended that not the quinine, but the morbid conditions requiring its use, caused the abortions in these cases. The cases in several instances seemed rather against him. But in the above mentioned case, it should be noted that the lady, six months advanced in a first pregnancy, had within a few days made several long rides on horseback and taken other rather severe exercise, had been much troubled with vomiting and was having two chills a day; enough, surely, to induce abortion without the aid of quinine.

What is the experience of others in regard to the parturifacient properties of quinine? Dr. Porter does not seem to have suspected it in the case quoted from his report. The extract contains all he says about the case, his object being merely to show the efficacy of quinine and pepper tea in congestive intermittent fever.

Some three weeks ago, I exhibited quinine in full doses, to a delicate, sensitive lady, *enciente* six months, having intermittent fever with *slight discharge* and *pain about the uterus*. No miscarriage occurred. And I can say emphatically that abortion has never followed the use of quinine in any case in my practice.

Small-pox, measles, ague, pertussis, dysentery, and many other diseases, when severe, induce abortion, or, in the non-gravid uterus, sanguineous discharges. There are so many ways of producing abortion by disease or accident, that I cannot readily assent to so grave a charge against one of the most valuable agents of the materia medica, unless sustained by unmistakable evidence.

---

Since writing the above, a report "*On the Medical Topography and Diseases of Fort Gibson, Arkansas*," has come into my hands. During four years, the hospital was in charge of Surgeon Randall and Assistant Surgeons McCormick and Coolidge, the last being the reporter. The report states that "The annual average of intermittent fever for the four years under consideration, is found to be 238 per cent. . . . The aggregate mean strength of the command being 944 men. . . . There were 2252 cases of intermittent reported." Many modes of treatment were tried—that by large doses of quinine, 15 to 20 grains, affording in all cases the most satisfactory results in every respect. One paragraph, exactly to the point in question, runs thus: "In fevers occurring in pregnant women, the quinine was given more freely than in ordinary cases, it being deemed important to prevent a return of the paroxysm, in order to prevent abortion or miscarriage, a result never caused by the use of quinine." Well and truly said, and as apropos as if written expressly to answer my question. Quinine preventing a return of the paroxysm of intermittent, (perhaps the most fruitful source of abortion,) prevents miscarriage. Withholding the quinine, by allowing a weakening of the system and strengthening of the causes of abortion, permits the latter to occur. In other words, by delaying the remedy too long, the woman passes into a condition so strongly predisposed to miscarriage, that quinine is unable, or simply fails, to prevent its occurrence.

